# Design and Application of a Metamaterial Superstrate on a Bio-Inspired Antenna for Partial Discharge Detection through Dielectric Windows

**DOI:** 10.3390/s19194255

**Published:** 2019-09-30

**Authors:** George Victor Rocha Xavier, Alexandre Jean René Serres, Edson Guedes da Costa, Adriano Costa de Oliveira, Luiz Augusto Medeiros Martins Nobrega, Vladimir Cesarino de Souza

**Affiliations:** 1 Post-Graduate Program of Electrical Engineering (PPgEE), Federal University of Campina Grande (UFCG), AprigioVeloso 882, Campina Grande 58429-900, Brazil; adriano.oliveira@ee.ufcg.edu.br; 2 Department of Electrical Engineering (DEE), Federal University of Campina Grande (UFCG), AprigioVeloso 882, Campina Grande 58429-900, Brazil; alexandreserres@dee.ufcg.edu.br (A.J.R.S.); luiz.nobrega@dee.ufcg.edu.br (L.A.M.M.N.); 3 Companhia Hidrelétrica do São Francisco (Chesf), Grisbert de Oliveira Gonzaga Street, Campina Grande 58418-105, Brazil; cesarino@chesf.gov.br

**Keywords:** bio-inspired design, metamaterial, gain enhancement, dielectric windows, partial discharges, printed monopole antenna, UHF sensors, monitoring

## Abstract

The adaptation of dielectric windows as metamaterial superstrate over a bio-inspired Printed Monopole Antenna (PMA) was evaluated in order to improve the detection sensitivity of Ultra High Frequency (UHF) sensors designed for Partial Discharge (PD) measurement. For this purpose, rectangular and circular Split Ring Resonators (SRR) structures were designed and evaluated aiming to achieve a metamaterial superstrate that improves the characteristics of the bio-inspired PMA as the gain, bandwidth, and radiation pattern. Measurements of the PMA with metamaterial superstrate were carried out in an anechoic chamber and compared to the simulations performed. The results show that the metamaterial superstrate insertion did not impact the original operating bandwidth, covering most of the characteristic frequency range of PD activity. Moreover, this insertion resulted in a mean gain enhancement of 0.7 dBi regarding the reference PMA, resulting in an antenna with better sensitivity for PD detection (mean gain of 3.61 dBi). The PMA-metamaterial set PD detection sensitivity was evaluated through laboratory tests with a point-to-plane PD generator setup and in field with measurements from a 230 kV current transformer. The developed PMA-metamaterial set was able to detect, successfully, the activity of PD for both tests, being classified as an optimized sensor for PD detection through dielectric windows.

## 1. Introduction

High voltage equipment insulating systems are subjected to stressful conditions, such as intense electric fields, chemical reactions, mechanical efforts, temperature variations, and several environmental phenomena. Due to the occurrence of these stresses, low magnitude electrical discharges that partially short-circuit the insulating material may occur. This phenomenon is defined as partial discharge (PD) [1].

The continuous action of the PDs can induce a significant degradation of the insulating material, which can eventually lead to a full dielectric breakdown and, consequently, equipment failure. Therefore, the continuous monitoring of PD activity in high voltage equipment is crucial to prevent the development of dielectric problems [2].

The most traditional method used for PD detection is the one established by the IEC 60270 standard [3], which allows the measurement of current pulses emitted during the occurrence of PDs. However, this method is considered highly invasive, since it requires an electrical connection with the monitored equipment through a coupling capacitor.

In order to overcome the practical limitations of the IEC 60270 standard method for online monitoring, researchers have been studying several alternative methodologies for PD detection [4,5,6,7,8]. Among these methods, the radiometric ones are highlighted due to their non-invasive nature, since there is no need of an electrical connection between the sensor and the monitored equipment. The radiometric or Ultra High Frequency (UHF) method consists of the detection of electromagnetic waves emitted by PD current pulses, which propagate through the insulation system of high voltage equipment in the UHF frequency range (300 MHz to 3 GHz). The UHF operation frequency range isolates the detection system from most electromagnetic interference in substations, such as power electronics switching and corona discharges, since the signals generated by these phenomena have components with significant energy for frequencies between 200 and 300 MHz [9]. Therefore, the UHF method is characterized as a non-invasive and interference-resistant technique, making it attractive for the continuous monitoring of high voltage equipment.

In order to improve the detection sensitivity of UHF sensors, researchers studied the spectral features of the PD radiation, concluding that, generally, most of the radiated energy is concentrated between 300 MHz and 1.5 GHz [9,10]. One of the pioneering papers regarding the application of UHF sensors in PD detection is presented in [11], which reports the detection and location of PDs in a 420 kV Gas Insulated Substation (GIS), attesting the efficiency of the UHF method. Over the years, other researches have proved the efficiency and reliability of the UHF method in GISs [12,13], and its application was extended to other high voltage equipment, mainly power transformers and high voltage cable connections [10,14,15].

**Figure 1:**
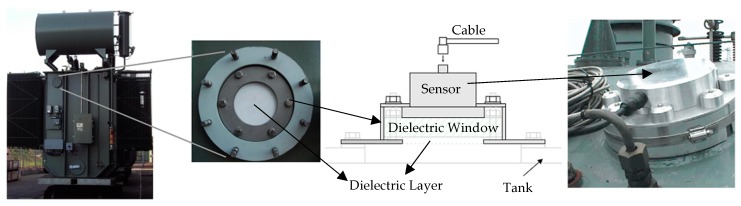
Sensor allocation through a dielectric window on a power transformer [14,17].

For power transformers and GIS, the allocation of UHF sensors is usually made through dielectric windows, as presented in Figure 1. Generally, a dielectric window can be defined as an opening covered by dielectric material on the equipment tank, providing a propagation way between the PD radiated waves and the installed UHF sensor [16].

The UHF sensors applied in dielectric windows for PD detection can assume several structures, such as loop electrode, disk electrode, conical coupler, and antennas (monopole, dipole, microstrip, and others) [18,19]. Among these sensors, printed monopole antennas (PMA) are highlighted for having desirable characteristics for practical applications, such as low cost, attractive radiation patterns, wideband, and ease of installation/construction [20]. However, PMA with traditional radiating element (patch) shapes designed to operate in UHF range would assume relatively large dimensions, limiting their use for practical applications in dielectric windows.

The operating frequency of PMA is directly related to the perimeter of the patch. The greater the perimeter, the lower the operating frequency. Therefore, techniques used for PMA miniaturization mainly seek optimized geometries that maximize the patch perimeter/area ratio. These optimized geometries can be addressed in the shapes of living beings developed in order to provide greater efficiency regarding the ability to survive. Hence, aiming to achieve a better radiating efficiency, bio-inspired antennas use the shapes of plants or animals as basis for their design [21,22,23,24,25,26].

In addition, in order to detect the inception of PD (low intensity pulses), the application of PMAs with high mean gain is required [27], making the evaluation of gain enhancement technique assimilation on PMAs designed for PD detection interesting.

Among the gain improvement techniques for PMA, the use of metamaterials stands out in the literature due to its characteristic of double negativity (DNG) for magnetic permeability and for electrical permittivity values [28,29,30,31]. The DNG feature results in the decreasing of material reflection and refractive indexes, acting as a converging lens that concentrates the electromagnetic waves in a given propagation medium, resulting in gain enhancement [32,33].

The application of metamaterials can be implemented in different ways, such as changes in the antenna patch [34], modifications in the ground plane [35], and applications as dielectric superstrates [36]. As can be seen from Figure 1, dielectric windows have a dielectric layer that naturally works as a superstrate. Thus, it is possible to take advantage of the dielectric layer to improve the gain of the antennas coupled to it by adding metamaterials in its structure. Therefore, the application of metamaterials in dielectric superstrates presents a greater practical feasibility for the detection of PD, since dielectric windows can be easily adapted for this purpose. In addition, the use of dielectric superstrates with metamaterials presents better gain enhancement results for broadband applications, as PD detection, in comparison to the application of modifications in the ground plane or antenna patch [37,38,39].

Therefore, the main objective of this paper is to design a metamaterial for gain enhancement that can be used as superstrate over a PMA developed for PD detection through dielectric windows. The PMA used as reference is a bio-inspired structure based on the *Jatropha mollissima* (Pohl) Baill leaf that was developed and presented in a previous work [26] and meets the size, bandwidth, and gain values requirements for PD detection through dielectric windows in high voltage equipment. Hence, this work aims to improve the PD sensitivity of detection of the structure presented in [26] through the application of a metamaterial superstrate representing a dielectric window, allowing a more accurate detection of PD inception (low intensity pulses). The designed metamaterial is composed by the combination of Split Ring Resonators (SRR) and Capacitive Load Strips (CLS). The bio-inspired PMA with metamaterial superstrate was subjected to bandwidth and gain measurement tests (in an anechoic chamber) and sensitivity tests of PD detection by means of comparative results with the IEC 60270 standard method connected to a PD generator laboratory setup. Finally, in order to evaluate the PD sensitivity of detection in practice, the bio-inspired PMA with metamaterial superstrate was tested in a substation to detect the PD activity on a 230 kV current transformer. 

## 2. Printed Monopole Antenna (PMA)

**Figure 2:**
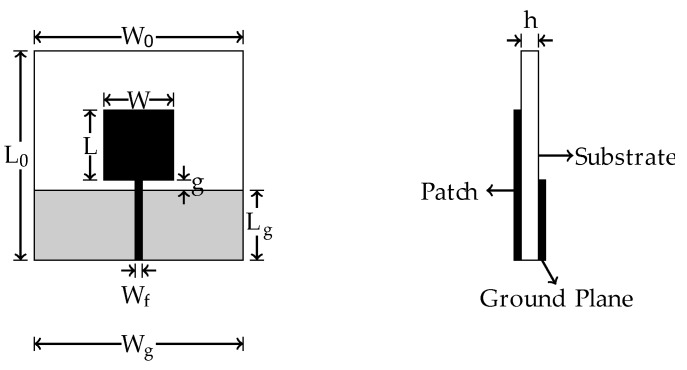
Printed Monopole Antenna (PMA) general structure.

PMA have a simple structure and great ease of construction and installation. In addition, features such as large bandwidth make this type of antenna suitable for PD application [26]. The general structure of a PMA is presented in Figure 2, in which *W_0_* and *L_0_* are the antenna length and width, respectively, *L* and *W* are the length and width of the patch, respectively, *L_g_* and *W_g_* are the length and width of the truncated ground plane, respectively, *W_f_* is the width of the transmission line, *g* is the distance between the patch and the ground plane, and *h* is the substrate thickness.

According to [21], the electric current density in a PMA is greater at the edges of the patch. Therefore, an increase in the patch perimeter would also increase the wavelength and, consequently, decrease the lower operating frequency. Hence, the lower frequency of the PMA operating band can be approximated by the patch perimeter, as presented in Equation (1) [21].

(1) $$f\left(GHz\right)=\;\frac{300}{p\sqrt{\varepsilon_{ref}}},$$

where *p* is the patch perimeter and *ε_re f_* is the relative permittivity of the dielectric, approximated as [21]:

(2) $$f\left(GHz\right)=\;\frac{300}{p\sqrt{\varepsilon_{ref}}}.$$

## 3. Metamaterials: Concept and Design

**Figure 3:**
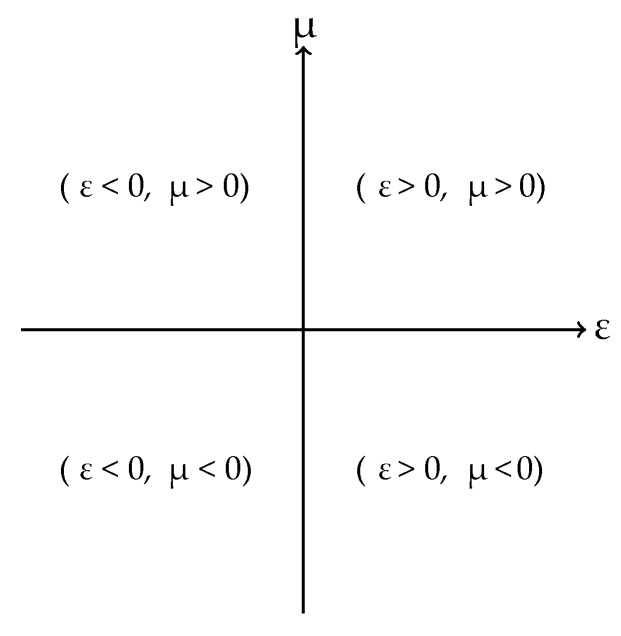
Material classification based on electrical permittivity and magnetic permeability values.

According to [28], from Maxwell equations, it is possible to classify the materials in four different classes according to their values of magnetic permeability (µ) and electrical permittivity (ε), as presented in Figure 3.

The first quadrant of Figure 3 (ε > 0, µ > 0) represents the materials in which the interaction between the electric and magnetic vectors (E and H, respectively) results in the propagation of a vector wave K with direction defined by the right hand rule, being denominated as RHM (Right Handed Materials). The RHM represents most of the materials commonly found in nature and presents a straight wave propagation mode [40].

The second quadrant (ε < 0, µ > 0) describes the electric plasmas in which evanescent waves propagate with higher efficiency [41]. The materials that are represented by this quadrant are known as ENG (Epsilon Negative). Similarly, the fourth quadrant (ε > 0, µ < 0) also presents greater propagation of evanescent waves, but for magnetic media. Therefore, the materials in this quadrant are called MNG (Mu Negative). Finally, the third quadrant (ε < 0, µ < 0) represents the metamaterials, or Double Negative (DNG) materials. Due to its double negative characteristic, the refractive index, calculated from Maxwell’s equations, also assumes negative values. The negative refractive index condition reflects in an interaction between E and H that results in the propagation of a K wave vector governed by the left hand rule. Thus, metamaterials can also be referred as LHM (Left Handed Materials) and have a reverse propagation mode regarding RHM. Besides the presentation of the characteristics described for ENG and MNG materials, the LHM have a negative refractive index that enables these materials to be able to concentrate incident electromagnetic waves in the medium in which they are applied. Hence, LHM can be defined as converging lenses that increase the focus of electromagnetic waves, both incident and evanescent waves, in a given dielectric medium.

The use of LHM as electromagnetic wave lenses has a useful practical application in microwave engineering, since a higher concentration of electromagnetic waves on the surface of an antenna, for example, can result in a considerable increase in its gain [42,43,44].

However, materials such as ENG, MNG, and LHM are not found in nature, requiring the manufacture of resonant structures that emulate such materials.

**Figure 4:**
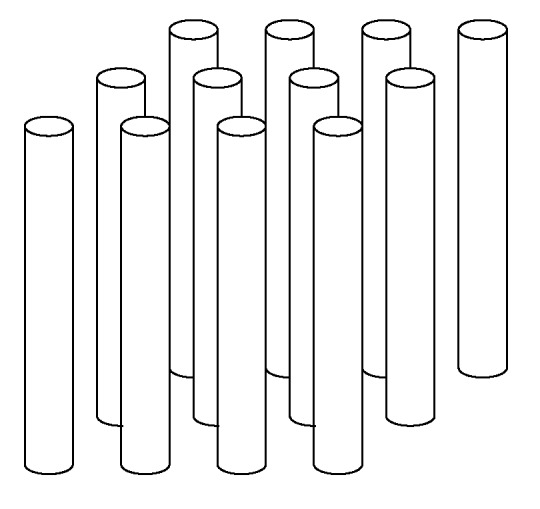
Example of Capacitive Load Strips (CLS) basic structure.

The behavior of an ENG-like material can be emulated from Capacitive Load Strips (CLS) that, for certain frequency ranges, may have higher electron concentrations and an increase in their effective mass, resulting in a negative electrical permittivity [29]. The basic structure of a CLS is presented in Figure 4.

For MNG-type materials, the emulation of their characteristics can be obtained from the use of a Split Ring Resonator (SRR). For specific frequency ranges, the electromagnetic interactions between the characteristic inductance of the rings and the capacitance present between their spacing results in high energy densities that characterize the material as MNG [30].

Therefore, from the joint application of CLS and SRR, it is possible to obtain a metamaterial that can be used as superstrate of antennas, resulting in the gain enhancement of these structures [36,39,45,46,47].

A more detailed physic-mathematical explanation regarding the functioning of SRR and CLS structures, as well as the interaction between them, can be found in [29,30,31,41,48,49].

The metamaterial operating frequency can be defined from the resonance frequency of the SRR geometry adopted. Although the SRR can be designed with several shapes [50,51,52], the circular and rectangular shapes represent the most adopted geometries in practice due to their lower complexity and ease of construction. Therefore, in this paper, only circular and rectangular SRRs were evaluated for the design of the proposed metamaterial.

**Figure 5:**
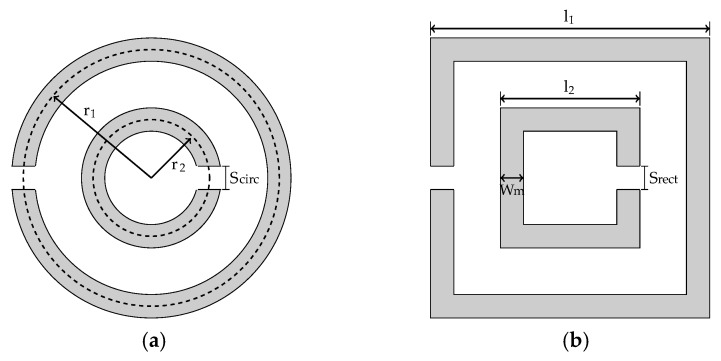
Examples of Split Ring Resonator (SRR) geometries: (**a**) circular; (**b**) rectangular.

The general structures of circular and rectangular SRR are presented in Figure 5, respectively.

For a circular SRR, the resonance frequency can be approximated by Equations (3) and (4) [53].

(3) $$f_{C1}=\;\frac{c}{2R_{1}\sqrt{\varepsilon_{ref}}}\;,\;f_{C2}=\;\frac{c}{2R_{2}\sqrt{\varepsilon_{ref}}},$$

(4) $$R_{1}=2\pi r_{1}-S_{circ}\;,\;R_{2}=2\pi r_{2}-S_{circ},$$

where *c* represents the speed of the light, *r_1_* and *r_2_* represent the external and internal mean radius length, respectively, and *S_circ_* is the existing gap in each ring with external and internal resonance frequencies defined by *f_C1_* and *f_C2_*, respectively. Similarly, for the rectangular SRR [53]:

(5) $$f_{R1}=\;\frac{c}{2L_{1}\sqrt{\varepsilon_{ref}}}\;,\;f_{R2}=\;\frac{c}{2L_{2}\sqrt{\varepsilon_{ref}}},$$

(6) $$L_{1}=4l_{1}-S_{rect}-4W_{m}\;,\;L_{2}=4l_{2}-S_{rect}-4W_{m},$$

where *l*_1_ and *l*_2_ represent the external and internal side length, respectively, and *W_m_* is the width of each resonance ring with external and internal resonance frequencies defined by *f_R_*_1_ and *f_R_*_2_, respectively.

In order to verify if the designed SRR–CLS set represents a metamaterial (ε < 0, µ < 0), the ε and µ values can be estimated from the refraction index (*η*) and impedance (*z*) values, as presented in Equations (7) and (8), respectively [54].

(7) $$\varepsilon =\frac{\eta }{z}\;,$$

(8) µ=ηz,

where *z* and *η* can be defined in terms of coefficients of reflection (*S*_11_) and transmission (*S*_21_) of the designed set, as presented in Equations (9) and (10), respectively [54].

(9) $$z=\;\pm \sqrt{\frac{{\left(1+S_{11}^{2}\right)}^{2}-S_{21}^{2}}{{\left(1-S_{11}^{2}\right)}^{2}-S_{21}^{2}}},$$

(10) $$\eta =\;\frac{1}{kl}\left\{Re\left[ln\left(e^{inkl}\right)\right]-Im\left[ln\left(e^{inkl}\right)\right]\right\},$$

in which, the *S*_11_ and *S*_21_ values are represented by its respective magnitude and phase components, *Re*[] and *Im*[] represent the real and imaginary components, respectively, *k* represents the wave number, *l* the longest metamaterial unit cell length, and *e^inkl^* is defined as [54]:

(11) $$e^{inkl}=\frac{S_{21}}{1-S_{11}\frac{z-1}{z+1}}.$$

## 4. Material and Methods

The applied methodology was divided in three different stages: computational procedures, laboratory experimental tests, and field application.

In the computational procedures, the software High Frequency Structure Simulator (HFSS) from ANSYS Electronics Desktop was used for all the simulation and design processes of the metamaterial (individually and positioned as superstrate). In all the simulations, the substrate is a low-cost fiberglass (FR-4) with ε_r_ = 4.4, *h* = 1.6 mm and loss tangent (δ) equal to 0.02. Moreover, the simulations were performed for the frequency of 200 MHz up to 1600 MHz with a discrete sweep type with 201 points. The solution frequency was centered in 1 GHz (central frequency for the designed antenna) with a maximum number of passes and Delta S equals to 6 and 0.02, respectively. 

The simulated PMA and metamaterial bandwidths were defined as the entire frequency range at which the reflection coefficient values were below the −10 dB threshold. In addition, the gain values were also extracted for the individual PMA and the set PMA-metamaterial in order to evaluate the gain enhancement provided by the metamaterial application as superstrate on the bio-inspired designed PMA. Lastly, the radiation patterns were obtained through simulations in order to evaluate if the metamaterial superstrate application resulted into distortions in the original bio-inspired PMA patterns.

In the experimental procedures, the designed PMA and the set PMA-metamaterial were built and subjected to comparative measurements of reflection coefficient and gain in an anechoic chamber in order to reduce the effect of external interferences and signal reflections during the tests. Then, the set PMA-metamaterial was used in a PD generator setup in order to estimate the PD detection sensitivity of the set regarding IEC 60270 standard method.

Finally, the PMA-metamaterial set was subjected to field application for the detection of partial discharges in a 230 kV Current Transformer (CT) from a Brazilian substation, Mussuré II (Chesf—Companhia Hidro Elétrica do São Francisco). 

In the following subsections, the computational procedures, laboratory experimental tests, and field application are detailed.

### 4.1. Bio-Inspired PMA Design

**Figure 6:**
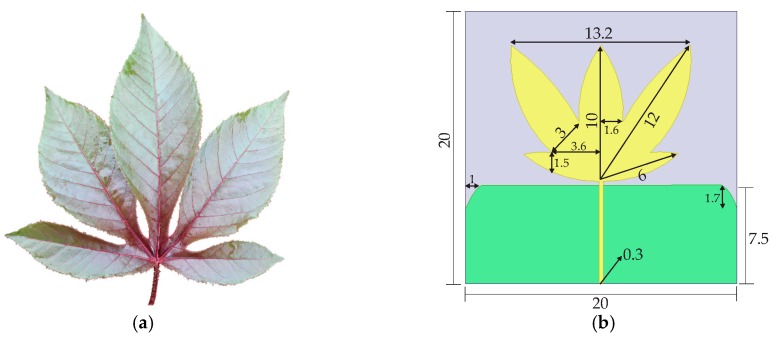
Bio-inspired PMA: (**a**) *Jatropha mollissima* (Pohl) Baill leaf; (**b**) details of the final designed model (all the dimensions are in centimeters) [26].

From the PMA model presented in Figure 2 and Equations (1) and (2), the bio-inspired PMA based on the *Jatropha mollissima* (Pohl) Baill leaf was obtained in [26]. The final dimensions of the designed bio-inspired PMA in [26] and a photograph of the *Jatropha mollissima* (Pohl) Baill leaf are presented in Figure 6.

### 4.2. Metamaterial Unit Cell Simulations

Through Equations (3)–(6) and dimensional fine adjustments, the resonance frequencies of the designed metamaterials unit cells were adjusted to present the DNG feature for the bandwidth within the PD activity frequency range (300–1500 MHz).

**Figure 7:**
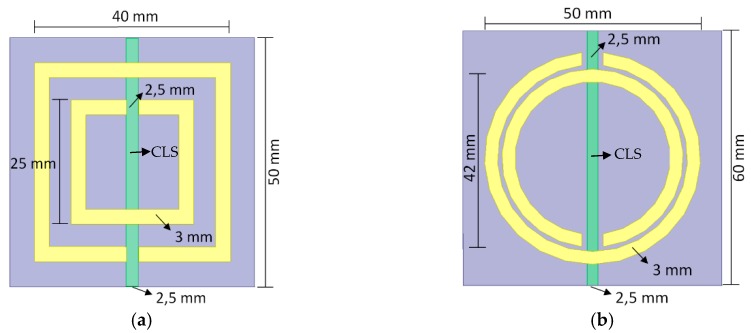
SRR models for each metamaterial unit cell (the green strips are the CLS structures): (**a**) rectangular; (**b**) circular.

The final dimensions for the designed rectangular and circular SRR-CLS metamaterial unit cells are presented in Figure 7, respectively.

**Figure 8:**
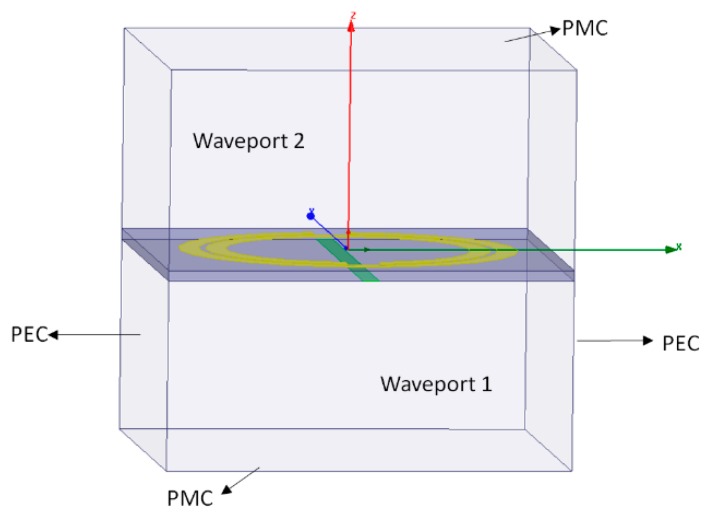
Boundary conditions for the simulated SRR-CLS models.

In order to characterize the models presented in Figure 7 as metamaterials, each model was simulated individually before the application as superstrate on the PMA. For the unit cells evaluation in electric and magnetic terms (ε and µ), special boundary conditions were established as presented in Figure 8, in which each metamaterial unit cell model is surrounded by perfect electric conductor walls (PEC) and perfect magnetic conductor walls (PMC) in parallel and orthogonal orientations regarding the *Z* axis, respectively. The unit cell model excitation is performed by two waveports. In this way, it is possible to acquire the *S*_11_ and *S*_21_ values used for the calculation of ε and µ, as presented in Equations (5)–(9). From the ε and µ values, it was possible to classify the unit cells as metamaterials (ε < 0 and µ < 0).

### 4.3. Metamaterial Superstrate Simulations

**Figure 9:**
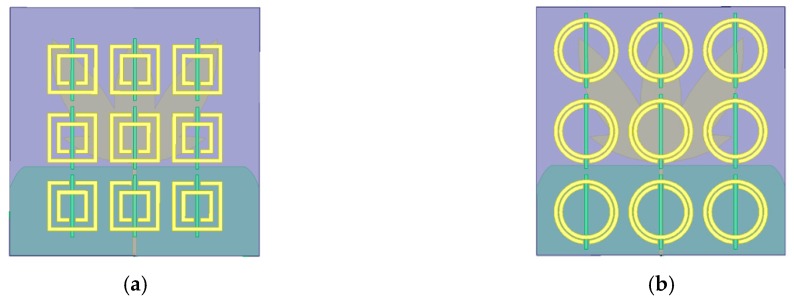
Metamaterial superstrates positioned over the bio-inspired PMA: (**a**) rectangular; (**b**) circular.

After the characterization as metamaterials, the simulated metamaterial unit cells were positioned in a 3 × 3 array as superstrates over the bio-inspired PMA as presented in Figure 9.

The superstrate height was adjusted so that the reflection coefficient values of the bio-inspired PMA were minimally impacted. The optimal superstrate height was defined as 135 and 125 mm for the rectangular and circular SRRs, respectively. In addition, the final array arrangement was also adjusted so that the gain values provided by the metamaterial superstrates were maximized. These adjustments consisted in some physical parameter variations, such as, the interconnection or not between the CLS of each unit cell, the adjustment in the horizontal and vertical spacing between the cells, the number of cells, and the position of the array regarding the PMA. Then, the horizontal and vertical spacing between the SRR unit cells were defined, respectively, as 10 and 15 mm, for the rectangular SRR superstrate, and as 8 and 14 mm, for the circular SRR superstrate. Moreover, the CLS of both unit cell designs were separated by a 5 mm gap.

**Figure 10:**
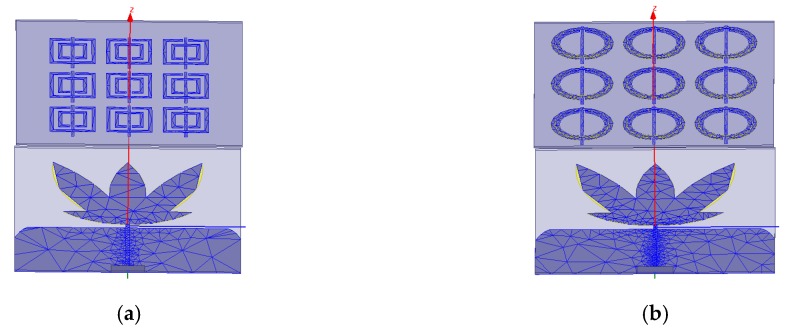
Meshing plot of the structures presented in Figure 9: (**a**) rectangular; (**b**) circular.

In addition, in order to attest that the gain improvement was actually coming from the inserted metamaterial structures and not from the dielectric material adopted as superstrate (FR-4), the bio-inspired PMA was also simulated with only the dielectric material as superstrate at similar height. After the application of the simulation settings presented at the beginning of this section in Figure 9 models, the following meshing presented in Figure 10 was obtained.

### 4.4. Reflection Coefficient and Gain Measurements

**Figure 11:**
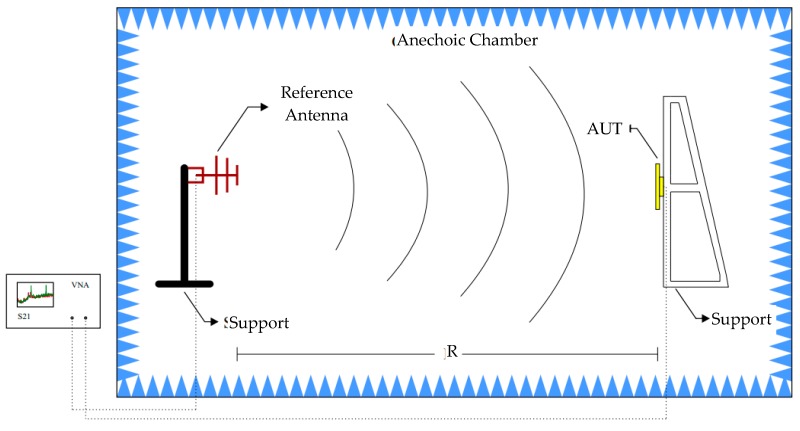
Schematic of the experimental setup used in the gain measurement test.

The reflection coefficient values were obtained through an ENA E5062A network analyzer from Agilent Technologies. For the gain measurements, an experimental setup composed by a reference antenna (Hyperlog 30100X: 380 MHz–10 GHz), with a 4.5 dBi mean gain, and the antenna under test (AUT) positioned beyond the far field distance (R) as presented in Figure 11.

The selected far field distance was defined regarding the maximum operation frequency of PD main activity range in oil, i.e., 1500 MHz [9,10]. Therefore, from the final bio-inspired PMA dimensions and the far field equations presented in [20], the value of R was defined as 1.0 m.

From the measured values of transmission and reflection coefficients and reference antenna gain (*G_R_* = 4.5 dBi), the designed bio-inspired PMA gain (*G_D_*) was calculated according to the adapted Friis equation [20]:

(12) $$G_{D}=G_{R}+{\left|S_{21}\right|}_{aut}^{2}-{\left|S_{21}\right|}_{ref}^{2}+\left(1-{\left|S_{11ref}\right|}^{2}\right)-\left(1-{\left|S_{11aut}\right|}^{2}\right)\;\;\left(dB\right)\;,$$

where, *S*_21aut_ and *S*_21ref_ represent the transmission coefficient of the antenna under test and reference antenna, respectively, and *S*_11aut_ and *S*_11ref_ represent the reflection coefficient of the antenna under test and reference antenna, respectively.

**Figure 12:**
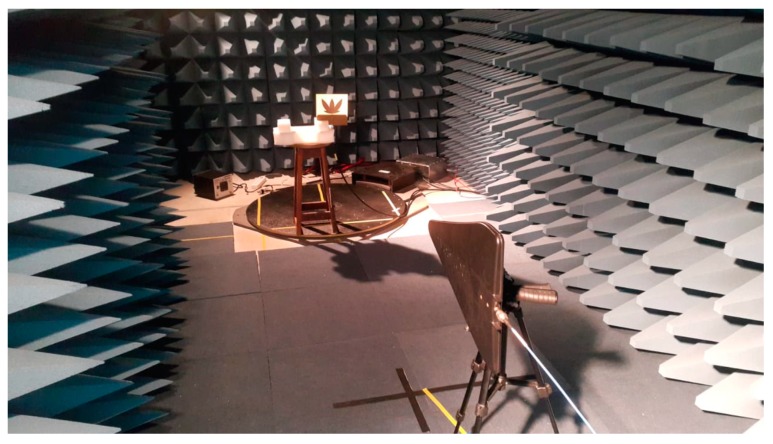
Photograph of the gain measurement test experimental setup.

For the application of Equation (12) in the gain estimation, it was necessary to assess the performance of two measurements using the schematic presented in Figure 8. The first measurement was performed using two identical reference antennas (Hyperlog 30100X), one positioned at the AUT distance and the other at the reference antenna position. In this way, it was possible to obtain the reference transmission coefficient (*S*_21ref_). The second measurement was performed with the PMA positioned at the AUT distance, allowing the obtaining of the *S*_21aut_. A photograph of the schematic shown in Figure 11 is presented in Figure 12.

The PMA sensitivity was tested by checking the criteria established by [27] for PD detection applications, which recommends that PMAs must present a mean gain higher than 2 dBi for their respective operating bandwidth.

### 4.5. PD Detection Sensitivity Tests

The experimental procedure applied for the generation and detection of PD consisted of a gradual increase of voltage, by means of a regulating transformer, until PD activity was generated in the PD source and detected by the bio-inspired PMA and the IEC 60270 standard method.

**Figure 13:**
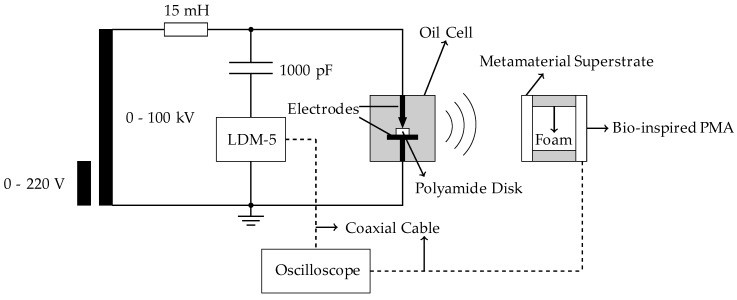
Experimental setup applied for Partial Discharge (PD) measurement.

The bio-inspired PMA was positioned at the selected far field distance (1.0 m) from the PD source. In order to represent typical internal partial discharge signals presented in high voltage equipment, an oil cell composed by a point-to-plane electrode configuration spaced by a dielectric (polyamide disk), was used as PD generator. The standard method is represented by a coupling capacitor (1000 pF) and a resonant circuit (LDM–5) as presented in Figure 13.

The PD pulses were acquired using a Keysight oscilloscope DSO90604A with 6 GHz bandwidth, 20 Gsa/s sampling rate, 70 ps rise time, and four analog channels.

In order to assess the antenna PD detection sensitivity in terms of apparent charge, all the calibration procedures established by the IEC 60270 standard were executed before the voltage application tests. For this, the LDC-5 calibrator from Doble Lemke was connected to the PD generator terminals and the pulses were collected by the LDM-5 system. The PD generator calibration apparent charge results are presented in Table 1.

Since the results presented in Table 1 show an approximately linear relation between the apparent charge and the measured voltage values, it is possible to correlate the apparent charge PD values, generated by the oil cell, with the voltage values measured by the IEC 60270 standard method. Then, the antenna PD detection sensitivity in terms of apparent charge can be evaluated. Calibration procedures for radiometric free-space, such as presented in [55], can also be used for this purpose.

### 4.6. PD Detection in a 230 kV Current Transformer

**Figure 14:**
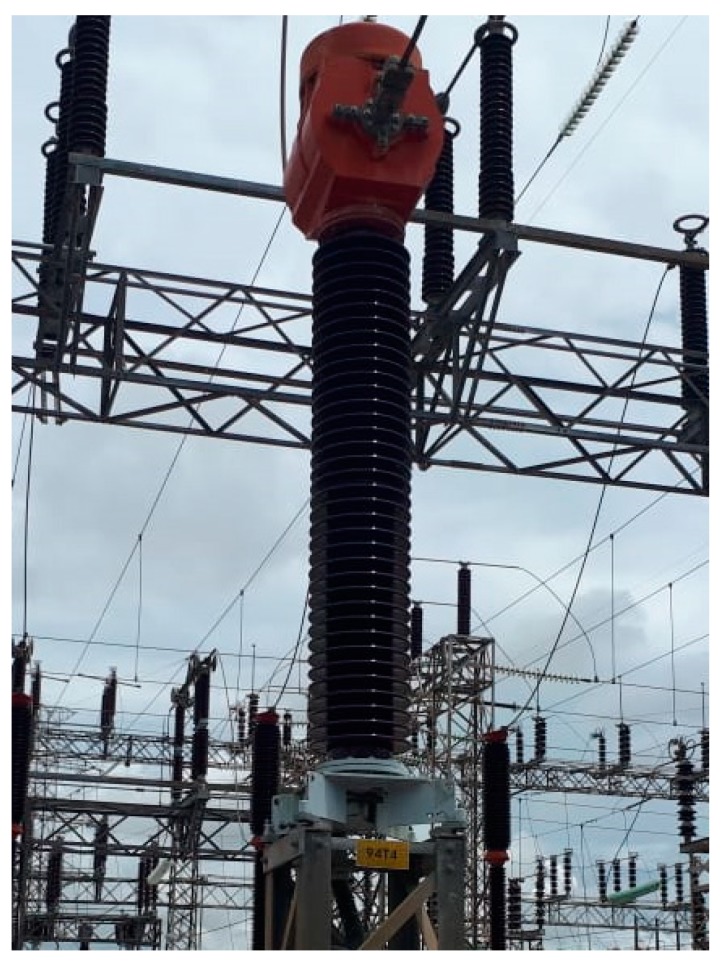
The 230 kV Current Transformer evaluated in the field application tests.

The equipment selected for the field application tests was a 230 kV Current Transformer (CT), presented in Figure 14.

Previous routine tests performed by the maintenance personnel, such as power factor tests, indicated CT insulating system degradation, pointed out the possibility of high partial discharges activity in this equipment. Therefore, this CT was selected for the field application, since a high partial discharge activity was expected. Such as for the PD sensitivity tests, the PD pulses were acquired in time domain. However, a Tektronix oscilloscope TDS5104B with 1 GHz bandwidth, 5 Gsa/s sampling, and four analog channels, was used.

According to [1,56], a PD signal can be identified by the pulse location regarding a reference voltage signal. Usually, the PD signals occur in the phase values corresponding to the semicycle transitions of a sinusoidal reference voltage. In addition, the PD pulses present a relative periodicity with a phase displacement of 180° between each pulse. Therefore, in order to evaluate if the signals emitted by the 230 kV CT and detected by the antenna were originated by PD, a signal generator was used in order to produce a sinusoidal reference voltage.

Moreover, the bio-inspired antenna without the metamaterial superstrate was used as reference in the field applications for comparative purposes. For this, both antennas were positioned simultaneously at the same distance from the CT. In addition, cables with the same length and attenuation were used. The signals were obtained simultaneously by triggering the oscilloscope. 

## 5. Results

The analysis of the results is divided into three subsections. The first is about the simulation results regarding the bio-inspired PMA, metamaterial unit cell, and superstrate. The second subsection is regarding the practical results regarding reflection coefficient and gain measurements, as well the PD detection sensitivity. Finally, Section 3 is regarding the field application of the designed bio-inspired PMA-metamaterial set on the PD detection in a 230 kV CT. 

### 5.1. Simulation Results

**Figure 15:**
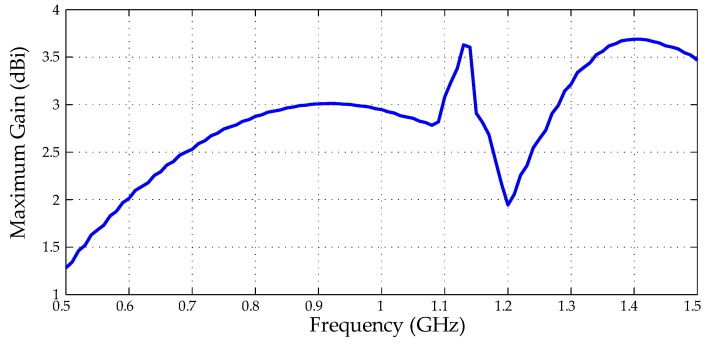
Simulated bio-inspired PMA maximum gain.

As previously mentioned, the bio-inspired PMA presented in [26] meets the size, bandwidth, and gain values requirements for PD detection through dielectric windows in high voltage equipment. However, although the PMA mean gain is higher than the 2 dBi criteria threshold, it can be verified in Figure 15 that the bio-inspired PMA presented maximum gain values lower to the recommended threshold for the frequency values near to the lower operating frequency (487 MHz). Therefore, in order to explore the dielectric windows for the PMA gain enhancement, metamaterial unit cells were simulated for the application as dielectric superstrates.

**Figure 16:**
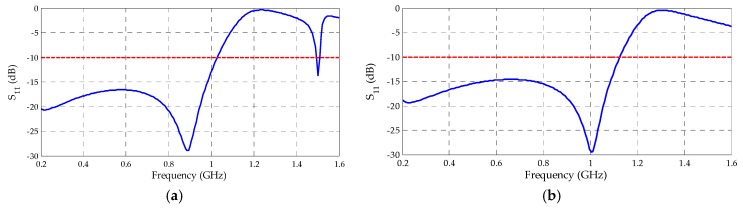
Metamaterial unit cells reflection coefficients: (**a**) circular; (**b**) rectangular.

The reflection coefficient results for the metamaterial unit cells with circular and rectangular SRR are presented, respectively, in Figure 16. 

Both the designed metamaterial unit cells presented bandwidths within the PD activity frequency range and for the frequency values that presented lower gain results for the bio-inspired PMA. The circular and rectangular unit cells presented operating bands up to 1033 and 1117 MHz, respectively.

**Figure 17:**
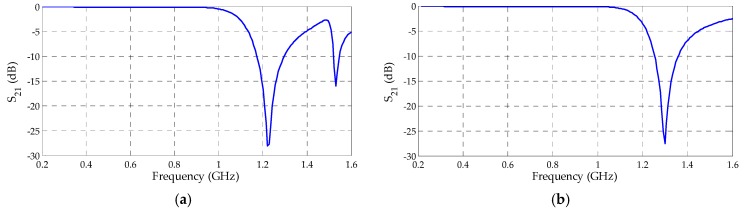
Metamaterial unit cells transmission coefficients: (**a**) circular; (**b**) rectangular.

**Figure 18:**
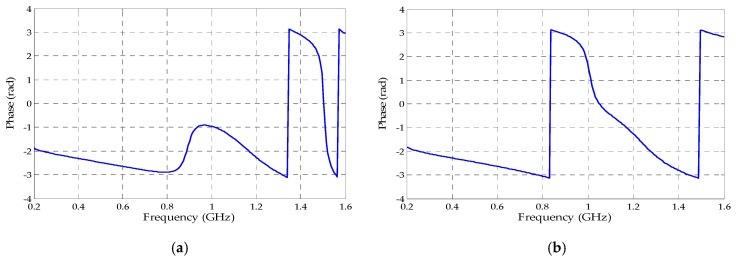
Reflection coefficient phases for the metamaterial unit cells: (**a**) circular; (**b**) rectangular.

**Figure 19:**
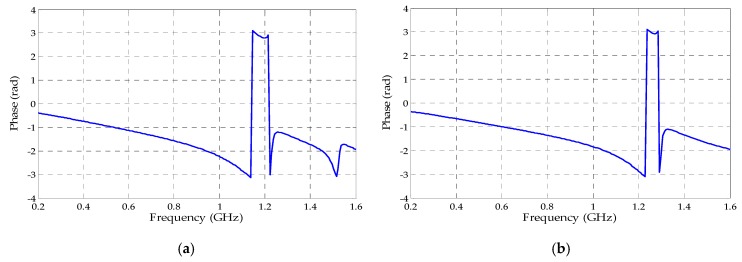
Transmission coefficient phases for the metamaterial unit cells: (**a**) circular; (**b**) rectangular.

**Figure 20:**
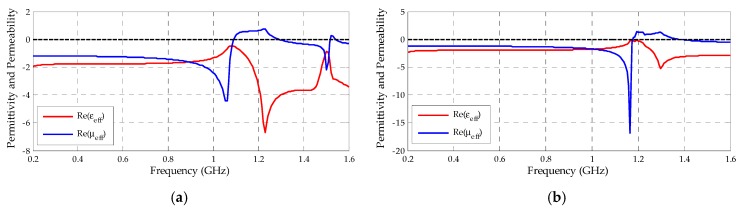
Metamaterial unit cells µ and ε values: (**a**) circular; (**b**) rectangular.

Besides the reflection coefficient, the transmission coefficient values were also obtained as presented in Figure 17. Therefore, through the magnitude and phase values ( Figure 18 and Figure 19) extracted from both coefficients (reflection and transmission), it was possible to calculate the permittivity (ε) and permeability (µ) values through Equations (5)–(9). The real values of µ and ε for both metamaterial unit cells are presented in Figure 20.

From Figure 20, it can be verified that both metamaterial unit cells presented double negative characteristic of ε and μ for almost the entire range of PD activity, with the exception of the bands corresponding to 1190–1350 MHz for the rectangular SRR and 1100–1300 MHz for the circular SRR, where the values of μ assume positive magnitudes. Therefore, both the SRR unit cells were classified as metamaterials applicable as superstrate for the designed bio-inspired PMA.

**Figure 21:**
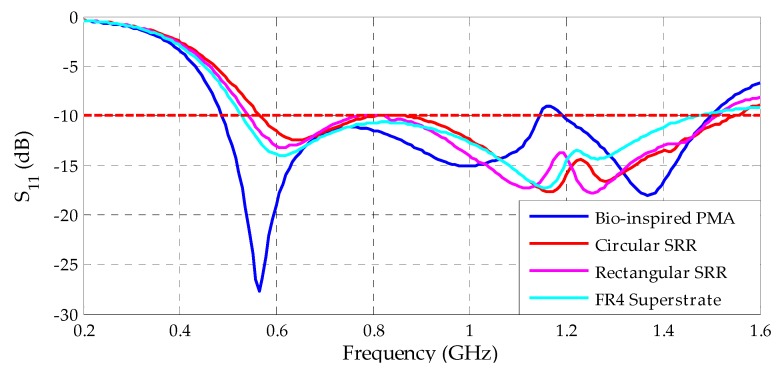
Comparison between the reflection coefficients of the simulated structures.

After the classification as metamaterials, the SRR unit cells were positioned as superstrates, as presented in Figure 9 and described in Section 4. The first analysis regarding the set PMA-Metamaterial is concerning the influence on the reflection coefficient due the application of the superstrate over the bio-inspired PMA. The results for this analysis are presented in Figure 21.

From Figure 21, it can be verified that the superstrate application, with or without the presence of the metamaterial unit cells, resulted in shifts in the lower operating frequency of the bio-inspired PMA. For the FR4 superstrate (without metamaterial), a displacement of 42 MHz can be observed, resulting in a 5.2% operating bandwidth reduction regarding the reference bio-inspired PMA (487–1497 MHz). For the metamaterial unit cells, the displacements verified for the lower operating frequency were 56 and 77 MHz for the rectangular and circular SRRs, respectively. In addition, the metamaterial application also resulted in an increase of the upper frequency for both SRR geometries, 22 MHz for the rectangular and 64 MHz for the circular, resulting in a 3.9% and 1.8% operating bandwidth reduction, respectively, regarding the reference bio-inspired PMA.

Moreover, the application of superstrates resulted in higher reflection coefficient values regarding the reference bio-inspired PMA for the frequency range of 1050–1300 MHz, resulting in an entire operating bandwidth with reflection coefficient values under the −10 dB threshold. Therefore, the superstrate addition had a positive influence, since the coefficient reflection values were improved, and no significant bandwidth changes were observed.

**Figure 22:**
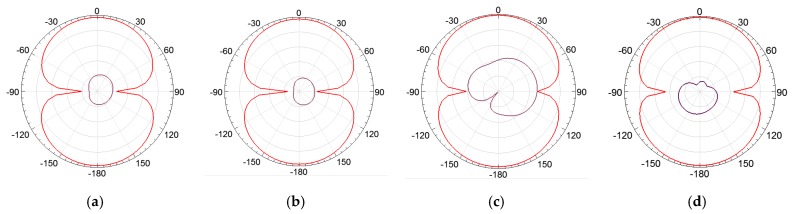
E-plane co-polarization (red) and cross-polarization (purple) radiation patterns for 487 MHz: (**a**) reference bio-inspired PMA; (**b**) FR4 Superstrate; (**c**) rectangular SRR; (**d**) circular SRR.

**Figure 23:**
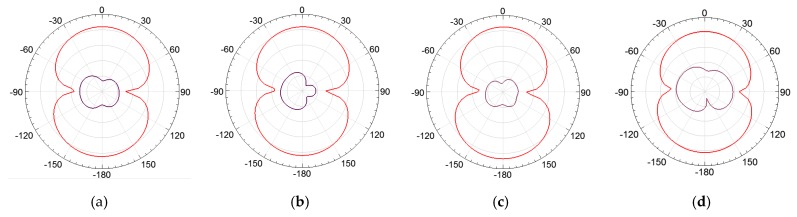
E-plane co-polarization (red) and cross-polarization (purple) radiation patterns for 992 MHz: (**a**) reference bio-inspired PMA; (**b**) FR4 Superstrate; (**c**) rectangular SRR; (**d**) circular SRR.

**Figure 24:**
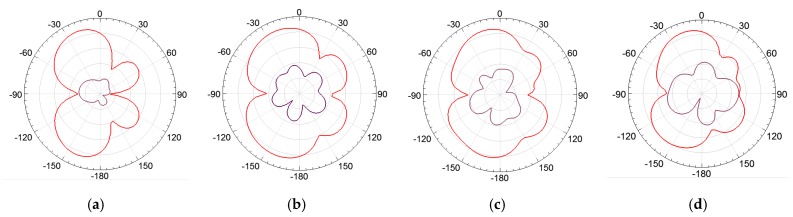
E-plane co-polarization (red) and cross-polarization (purple) radiation patterns for 1497 MHz: (**a**) reference bio-inspired PMA; (**b**) FR4 Superstrate; (**c**) rectangular SRR; (**d**) circular SRR.

In order to assess the superstrate’s influence on the bio-inspired PMA radiation patterns, the lower, central, and upper frequency values were simulated and presented in Figure 22, Figure 23 and Figure 24.

From Figure 22, Figure 23 and Figure 24, it can be verified that the superstrate addition without SRRs (only FR4) did not significantly change the radiation patterns regarding the reference bio-inspired PMA. However, radiation pattern distortions were verified for the upper operating frequencies for both SRR superstrates, as presented in Figure 24c,d.

**Figure 25:**
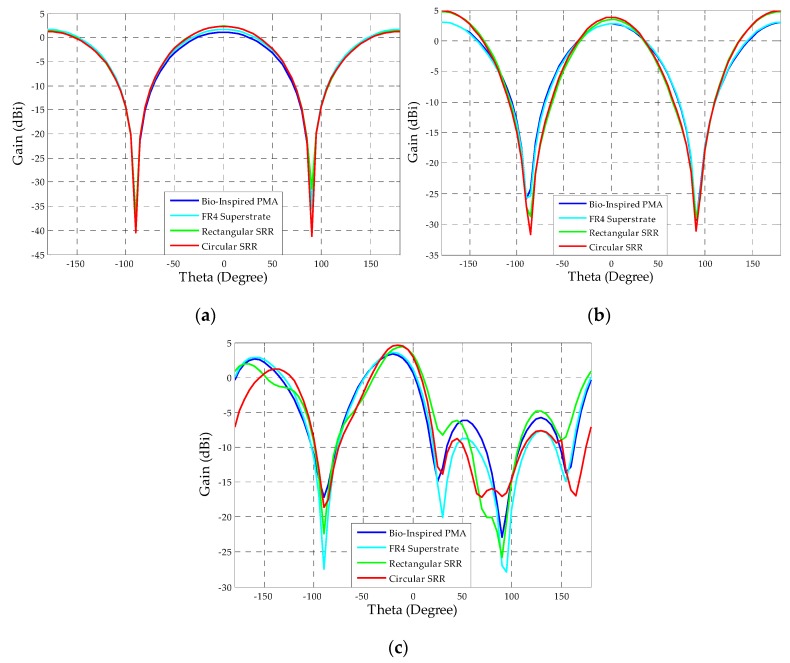
Rectangular plots of the E-plane radiation patterns for the frequencies of (**a**) 500 MHz; (**b**) 900 MHz; (**c**) 1500 MHz.

Moreover, from the obtained radiation patterns, it can be verified that the antenna with metamaterial is suitable for dielectric window application, since the most of maximum radiation values are concentrated in the direction of coupling of the PMA to the dielectric window, i.e., 0° direction as presented in the Figure 25. In addition, considering that the PMA coupled to the dielectric window is back shielded against external interferences, the symmetrical back lobes presented in the radiation patterns also represent an advantage in the application for PD detection, since the back lobe allows the detection of the PD radiated signals reflected by the shielding, enhancing the PMA sensitivity of detection [26].

**Figure 26:**
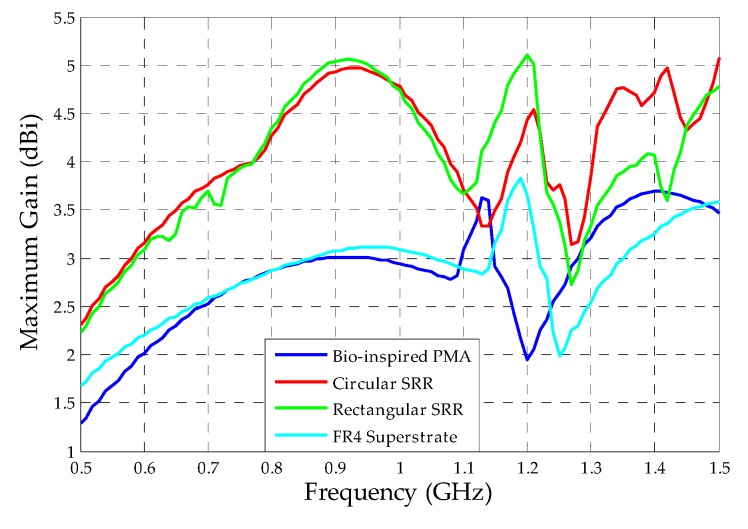
Comparison between the maximum gain values of the simulated structures.

Finally, from the extracted radiation patterns, the maximum gain values along the operating frequency for each simulated structure were obtained, as presented in Figure 26.

The application of both designed superstrates resulted in maximum gain values almost increased in double regarding the reference bio-inspired PMA for the frequency range of 500–1000 MHz. In addition, the circular SRR superstrate also demonstrated significant gain improvement for the frequency range of 1300–1500 MHz, probably due to the strong DNG feature presented for this frequency range, as presented in Figure 14.

Before the superstrate application, the reference bio-inspired PMA presented gain values higher than 2 dBi only for frequencies above 600 MHz. With the metamaterial addition, the gain values were higher than 2 dBi for the entire antenna operating frequency (500–1500 MHz).

The calculated mean gain values for each simulated structure are presented in Table 2.

From Table 2, it can be noticed that the metamaterial application as superstrate resulted in a maximum mean gain improvement higher than 1 dBi, regarding the reference bio-inspired PMA, for both SRR geometries chosen. Although the circular SRR structure presented a slightly higher mean gain value (4.07 dBi) in comparison to the rectangular geometry (3.96 dBi), the reflection coefficient results for the rectangular SRR presented lower bandwidth impacts for PD application than the circular one. In addition, the rectangular SRR presented lower radiation patterns distortions than the circular one for the upper frequencies, mainly in the back lobe direction, as presented in Figure 24 and Figure 25. Therefore, since symmetrical back lobes are desirable for PD detection through dielectric windows, the rectangular SRR was considered the fittest structure for the experimental PD tests presented in the following section.

### 5.2. Laboratory Experimental Results

**Figure 27:**
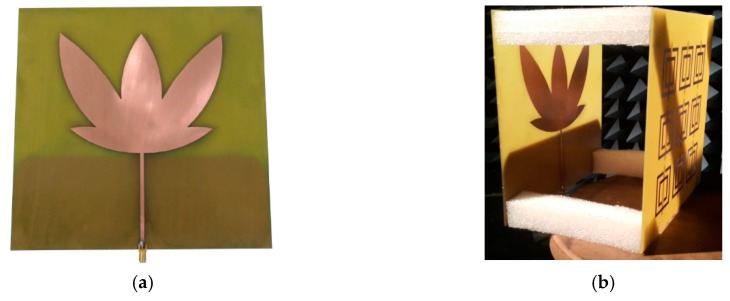
Manufactured bio-inspired PMA: (**a**) without metamaterial superstrate [26]; (**b**) with metamaterial superstrate.

As mentioned in the previous subsection, the simulated reflection coefficients, radiation patterns, and mean gain appointed the rectangular SRR metamaterial superstrate as the fittest structure for the experimental PD tests. Therefore, from the models presented in Figure 6b and Figure 9a, the bio-inspired PMA without and with the metamaterial superstrate were built, as presented in Figure 27.

**Figure 28:**
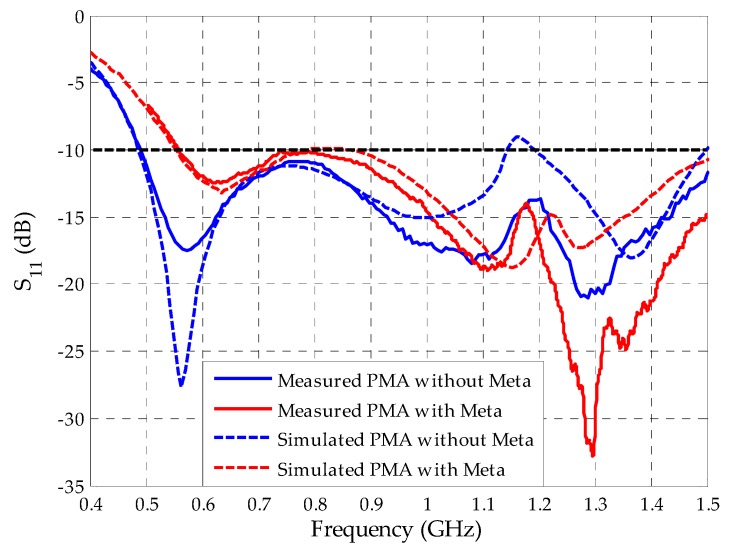
Measured and simulated reflection coefficients for the bio-inspired PMA with and without metamaterial superstrate.

The first experimental analysis for the manufactured antennas is regarding the reflection coefficients. In Figure 28 a comparison between the measured and simulated reflection coefficients for the bio-inspired PMA with and without the rectangular SRR metamaterial superstrate is presented.

Both measured and simulated reflection coefficient results presented similar behavior. For the bio-inspired PMA without metamaterial, the measured operating bandwidth started at 472 MHz up to values above the upper PD frequency of interest (1500 MHz). With the metamaterial, the lower frequency was displaced in 68 MHz (close to the 77 MHz simulated value) regarding the reference bio-inspired PMA, starting from 540 MHz until values above 1500 MHz. In addition, better reflection coefficient performance for the frequency range of 1.1–1.5 GHz was verified for the measured curve with metamaterial, as predicted by the simulations. The slight differences between the simulated and measured reflection coefficient behaviors can be attributed to PMA constructive aspects, such as minor discrepancies between the simulated FR4 and the one used in practice, as well the non-consideration of the SMA connectors during the simulation.

**Figure 29:**
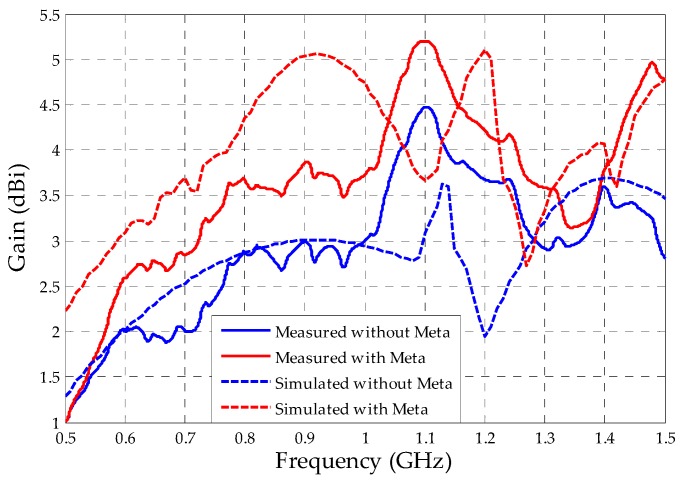
Measured and simulated gain for the bio-inspired PMA with and without metamaterial superstrate.

Through the procedures described in Section 4, the bio-inspired PMA gains with and without the metamaterial superstrates were measured. The comparison between the measured and simulated gains are presented in Figure 29.

From Figure 29, it can be observed that, although there is a discrepancy in the gain values, the behavior of the measured and simulated curves are in agreement. 

For both measured and simulated curves for the bio-inpired PMA without metamaterial, an increase in the gain values up to frequencies around 1.1 GHz can be verified, followed by a gain decrease up to 1.2 GHz and, again, a gain increase up to 1.4 GHz, followed by a new decrease tendency up to frequency values above 1.5 GHz. For both measured and simulated curves for the bio-inspired PMA with metamaterial, the same trend was verified, except for the frequencies above 1.4 GHz, in which the bio-inspired PMA with metamaterial presented a gain increase trend up to frequencies above 1.5 Ghz.

Besides the constructive aspects mentioned before, the differences between the measured and simulated values can be attributed to the conception of the simulated environment, which neglects some practical limitations faced during the measurements, such as the cables attenuation, the alignment between the reference antenna and the test antenna, and the losses in the far-field measuring system.

Even so, the gain enhancement provided by the metamaterial superstrate was according to that predicted by the simulations, presenting significant gain improvement for mostly the bandwidth of the bio-inspired PMA. The measured mean gain for the bio-inspired PMA without metamaterial was equal to 2.92 dBi, close to the simulated mean gain value presented in Table 2 (2.86 dBi). In addition, the bio-inspired PMA without metamaterial only presented gain values above the 2 dBi threshold for the frequencies above 700 MHz. With the metamaterial superstrate, the calculated mean gain was 3.61 dBi, resulting in an enhancement of 0.7 dBi regarding the bio-inspired PMA. Moreover, the application of the metamaterial superstrate resulted in an antenna with gain values above 2 dBi from 575 MHz up to 1.5 GHz, resulting in an antenna with high sensitivity for PD detection.

From the measured reflection coefficient and gain results presented, the bio-inspired PMA-metamaterial set can be considered fit for PD detection. Therefore, it was submitted to high voltage PD detection sensitivity tests.

**Figure 30:**
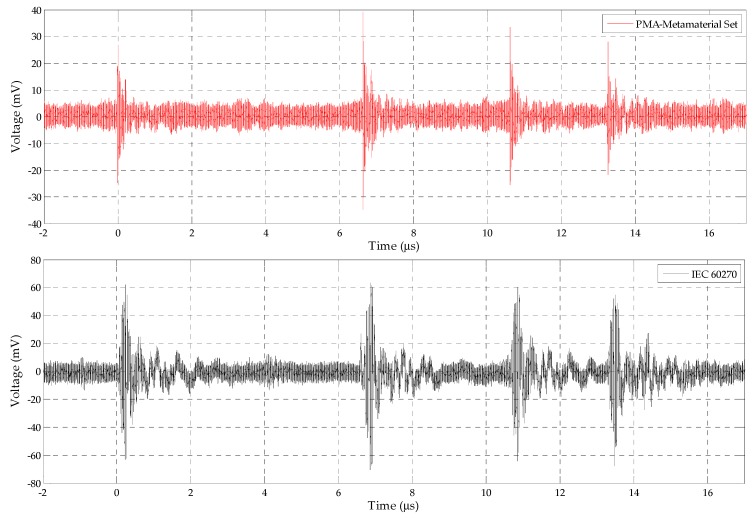
PD pulses sample detected by the bio-inspired PMA-metamaterial set and IEC 60270 standard method, respectively, for the application of 13.4 kV on the oil cell.

Through the setup presented in Figure 13, the PD activity was detected simultaneously by the bio-inspired PMA-metamaterial set and the IEC 60270 standard method for the application of 13.4 kV on the oil cell. Figure 30 presents one sample of the simultaneously detected PD pulses.

From Figure 30, it can be verified that the bio-inspired PMA-metamaterial set was able to detect all the PD pulses detected by the IEC 60270 standard method. In addition, the voltage measured at the antenna’s terminals presented values reduced by one-half compared to the standard method. This result is expected, since the radiated waves are susceptible to higher losses, such as reflections and propagating attenuations, than those in the standard method, directly connected with the oil cell. Nonetheless, the antenna sensitivity could be attested, since, according to Table 1, the detected PD pulses presented apparent charge values between 15 and 60 pC. This range of apparent charges is similar to the pC values detected in practice for the PD low intensity pulses related to the inception of an insulating problem in transformers oil, for example in [57,58,59].

**Figure 31:**
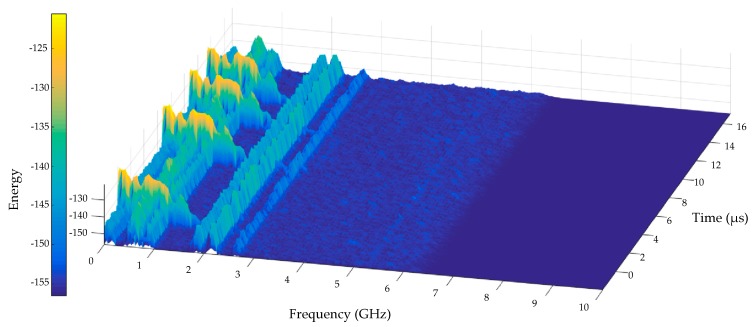
Spectrogram of the PD pulses sample detected by the bio-inspired PMA-metamaterial set presented in Figure 30.

In order to evaluate the frequency range of the detected PD pulses, a spectrogram was extracted from the PMA detected PD pulses shown in Figure 30 and presented in Figure 31. 

From Figure 31, it can be verified that the four PD pulses observed in Figure 23 were highlighted in the spectrogram, presenting higher concentrations of spectral energy for frequencies up to 1500 MHz. The obtained frequency range is in agreement with the values reported in [9,10,60] as the main range of interest for the monitoring of partial discharges in oil (300–1500 MHz). 

Therefore, besides the sensitivity (gain), the antenna operating bandwidth was also attested in the high voltage PD tests, since the bio-inspired PMA-metamaterial set was able to successfully detect all the main frequency range of energy concentration for PD pulses.

Hence, the bio-inspired PMA-metamaterial set was approved in the laboratory PD experimental tests, being able to be applied in PD measurements on substations, as presented in the following subsection. 

### 5.3. PD Detection in a 230 kV Substation

**Figure 32:**
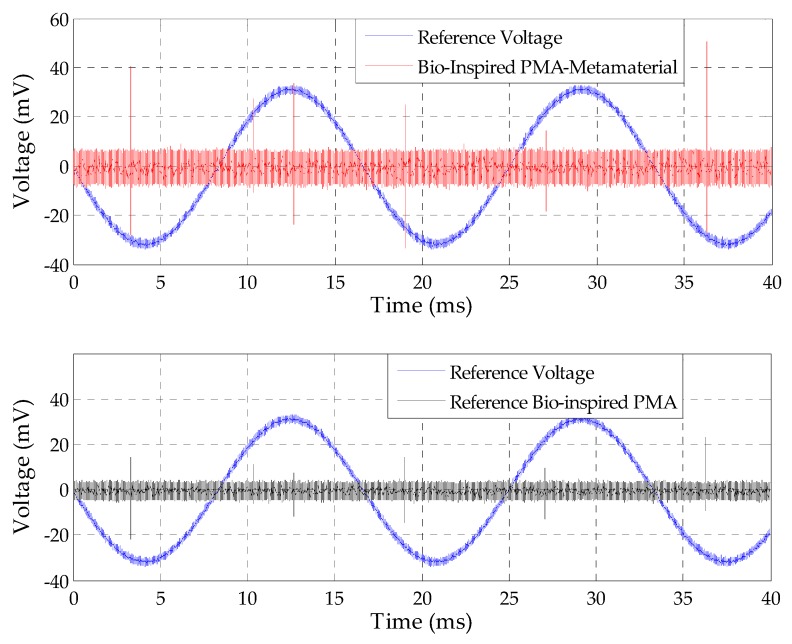
Comparison between the 230 kV Current Transformer (CT) PD pulses detected by the bio-inspired PMA with and without the metamaterial superstrate, respectively.

Applying the methodology presented in Section 4.6, PD measurements on a 230 kV CT were performed and the comparison between the PD pulses detected by the bio-inspired PMA-metamaterial set and by the reference bio-inspired PMA are presented in Figure 32.

From Figure 32, it can be verified that the detected signals presented PD features, since they occurred in the half-cycle transitions of the sinusoidal reference voltage and with a phase displacement approximately equal to 180° between each other. In addition, the bio-inspired PMA-metamaterial set presented higher detection sensitivity than the reference bio-inspired PMA for all of the detected PD pulses, presenting a signal with amplitudes almost doubled regarding the reference bio-inspired PMA.

As expected, according to previous measurements performed by the substation maintenance personnel, a considerable level of PD was detected in the 230 kV CT, attesting the sensitivity of the developed antenna in the practical detection of PD. 

Although the PMA presented high sensitivity for PD detection, it was not possible to estimate the PD apparent charge levels inside the equipment and detected by the antennas, since the calibration procedure on the 230 kV CT could not be performed due to practical limitations (coupling capacitor parallel connection). Therefore, at this point of the research, the CT operational condition diagnosis (low, medium, or critical level of PD) could not be estimated.

Nonetheless, the application of the developed bio-inspired PMA-metamaterial set in the CT PD measurement was done in order to attest its practical sensitivity in the PD detection. Hence, as future works, calibration, classification, and localization techniques will be applied over the proposed antenna in order to fully characterize it as a sensor applicable in the PD continuous monitoring and diagnosis, specially through dielectric windows improved by the application of metamaterials on its surface.

## 6. Discussion

Although there are more competitive antenna types that can provide higher gain than PMA, such as hornet and Vivaldi antennas, their use in PD detection is compromised due to their constructive aspects (mainly their length), since they are not suitable for installation in dielectric windows in power transformers or GIS. Despite the fact that PMA are not as efficient as another types of antennas, they still represent a good option for PD detection, since its large bandwidth, gain, radiation pattern and dimensions are still suitable for PD detection, as verified and shown on other studies in the literature [25,26,27,61,62,63,64,65,66]. 

As presented in this paper, the distance between the antenna and the metamaterial superstrate (dielectric window) was adopted in order to reduce the impact on the antenna's reflection coefficient. Still, even with the distance between the bio-inspired antenna and the metamaterial superstrate (dielectric window), the final structure has a volumetric dimension of 200 mm × 200 mm × 135 mm to accommodate an antenna with an operating bandwidth of 500–1500 MHz and average gain of 3.61 dBi, which represents an acceptable size for PD application and good PD detection sensitivity features as demonstrated experimentally during the paper and through comparisons with other results obtained in the literature [67,68,69,70,71,72,73]. If another type of antennas were used for this same bandwidth, their higher length would require a much larger dielectric window, becoming an impractical application. The dimensions of some more directional antennas (commercial and from other papers) with lower operating frequency equivalent to the proposed antenna (500 MHz) are presented in the Table 3. 

As can be seen from Table 3, although the good results of bandwidth and gain for PD detection application, all the other antennas present a much larger length than the proposed PMA-dielectric window set (135 mm). Moreover, by coupling the presented antennas over dielectric windows, we would have even larger dimensions for the antenna-dielectric window set than those presented in Table 3, becoming impractical for the application on PD detection in power transformers or GIS. Therefore, this reinforces the advantage of using PMA for the achievement of dielectric windows with smaller dimensions and higher ease of coupling on the structure of high voltage equipment.

## 7. Conclusions

In this paper, the representation of dielectric windows as a metamaterial superstrate over a bio-inspired antenna (based on the *Jatropha mollissima* (Pohl) Baill leaf) was proposed in order to improve the detection sensitivity of UHF sensors designed for PD measurement. During the simulation tests, both the selected SRR shapes (rectangular and circular) proved themselves efficient in the enhancement of the antenna detection sensitivity, presenting a mean gain improvement higher than 1 dBi compared to the structure without metamaterial. However, the rectangular SRR was selected for the laboratory tests due to the better relation gain/bandwidth/radiation pattern achieved. The designed bio-inspired PMA-metamaterial set presented potential to be used as a UHF sensor applied through dielectric windows for PD detection, since it meets the bandwidth (300–1500 MHz) and gain (>2 dBi) requirements according to the laboratory tests carried out in an anechoic chamber. This potential was attested in the PD detection tests, in which the developed sensor was able to detect several PD signals generated in laboratory, by the point-to-plane electrode configuration, and in practice, by a 230 kV CT. Although the PD apparent charge levels could not be estimated in practice through the signals detected by the antenna, the evaluation of the induced voltage in the antenna terminals can provide information about the PD activity evolution inside the equipment. Therefore, the proposed bio-inspired PMA-Metamaterial set can provide continuous, non-invasive, and relatively low-cost monitoring of PD activity by means of dielectric windows in high voltage insulating systems. 

The conclusions obtained from the results of this article can be summarized as follows:

(1) Both the SRR-CLS designed unit cells presented the double negative behavior (µ < 0, ε < 0) for almost all the main frequency range of PD activity (300–1500 MHz). Therefore, both the SRR unit cells were classified as metamaterials applicable as superstrate for the designed bio-inspired PMA;
(2) The application of a metamaterial superstrate resulted in displacements in the bio-inspired PMA operating frequency range, resulting in a 3.9% and 1.8% bandwidth reduction for the circular and rectangular SRRs, respectively;
(3) Although there was a bandwidth reduction regarding the bio-inspired PMA, the application of the both metamaterial superstrates resulted in higher reflection coefficient values for the frequency range of 1100–1500 MHz, in which some reflection coefficient values were lower than the threshold of −10 dB, improving the antenna power transmission/reception capacity for this frequency range;
(4) Radiation patterns distortions were observed at the upper bio-inspired PMA operating frequencies for both metamaterial superstrates, in which the rectangular SRR presented lower radiation patterns distortions than the circular one for the upper frequencies, mainly at the back lobe direction;
(5) The application of both designed superstrates resulted in maximum gain values almost increased in double regarding the reference bio-inspired PMA for the frequency range of 500–1000 MHz, resulting in a mean gain improvement higher than 1 dBi;
(6) The practical application of the rectangular SRR superstrate resulted in a UHF sensor with an operating bandwidth (540–1500 MHz) that covers 80% of the main frequency range of PD activity (300–1500 MHz);
(7) The measured mean gain for the bio-inspired PMA-metamaterial set was equal to 3.61 dBi, resulting in an enhancement of 0.7 dBi regarding the bio-inspired PMA (2.92 dBi) and representing a good detection sensitivity for PD application;
(8) In PD laboratory tests, the bio-inspired PMA-metamaterial set was able to detect apparent charges above 15 pC, generated in a point-to-plane electrode configuration in an oil cell;
(9) The bio-inspired PMA presented measured voltage values with half of the magnitude obtained from the IEC 60270 standard method, resulting in a high PD detection sensitivity for a radiometric based method;
(10) Lastly, the bio-inspired PMA-metamaterial set presented itself as effective in the practical PD detection application, since it was possible to detect a significant level of PD activity in a 230 kV substation CT.

As future works, the sensor will be improved through the application of calibration, classification, and localization techniques in order to fully characterize it as a PD UHF sensor applicable for equipment diagnosis.

## Figures and Tables

**Table 1:** Apparent charge calibration results for the PD generator used.

Apparent Charge (pC)	Voltage (mV)
20	25.6
100	121
500	584

**Table 2:** Simulated mean gain values for each simulated model.

Model	Maximum Mean Gain (dBi)
Bio-inspired PMA	2.81
FR4 Superstrate	2.83
Rectangular SRR	3.96
Circular SRR	4.07

**Table 3:** Comparison between the proposed PMA-Metamaterial set and other type of antennas.

Antenna	Bandwidth (GHz)	Gain (dBi)	Size (mm)
Proposed PMA-Metamaterial Set	0.5–1.5	3.61 (mean)	200 × 200 × 135 (depth)
[74] (Hornet Antenna)	0.5–8	3–17	418 × 318 × 645 (length)
[75] (Hornet Antenna)	0.5–3	4–11	440 × 290 × 350 (length)
[76] (Vivaldi Antenna)	0.5–6	3.07–7.7	120 (width) × 225 (length)
[77] (Vivaldi Antenna)	0.5–4	2–11	173 (width) × 299 (length)
[78] (Reference Antenna–Hyperlog 30100X)	0.4–10	4.5 (mean)	360 (width) × 640 (length)

## References

[1] Natrass D.A. (1988). Partial Discharge Measurement and Interpretation. IEEE Electr. Insul. Mag..

[2] Kreuger F.H., Gulski E., Krivda A. (1993). Classification of partial discharges. IEEE Trans. Electr. Insul..

[3] International Electrotechnical Comission (2000). High Voltage Test Techniques: Partial Discharge Measurements.

[4] Stone G. (2005). Partial discharge diagnostics and electrical equipment insulation condition assessment. IEEE Trans. Dielectr. Electr. Insul..

[5] Meira M., Ruschetti C.R., Álvarez R.E., Verucchi C.J. (2018). Power transformers monitoring based on electrical measurements. IET Gener. Transm. Distrib..

[6] Stone G.C. (2013). Condition monitoring and diagnostics of motor and stator windings—A review. IEEE Trans. Dielectr. Electr. Insul..

[7] Refaat S.S., Shams M.A. A review of partial discharge detection, diagnosis techniques in high voltage power cables. Proceedings of the 2018 IEEE 12th International Conference on Compatibility, Power Electronics and Power Engineering (CPE-POWERENG).

[8] Sahoo A., Subramaniam A., Bhandari S., Panda S.K. A review on condition monitoring of GIS. Proceedings of the 2017 International Symposium Electrical Insulating Materials (ISEIM).

[9] Tenbohlen S., Denissov D., Hoek S.M., Markalous S.M. (2008). Partial discharge measurement in the ultra high frequency (UHF) range. IEEE Trans. Dielectr. Electr. Insul..

[10] Judd M., Pryor B., Kelly S., Hampton B. Transformer monitoring using the UHF technique. Proceedings of the 11th International Symposium on High Voltage Engineering (ISH).

[11] Hampton B.F., Meats R.J. (1988). Diagnostic measurements at UHF in gas insulated substations. IEEE Proc..

[12] Masaki K., Sakakibara T., Murase H., Akazaki M., Uehara K., Menju S. (1994). On-site measurement for the development of on-line partial discharge monitoring system in GIS. IEEE Trans. Power Del..

[13] Judd M., Farish O., Pearson J. (1997). UHF couplers for gas-insulated substations: A calibration technique. IEEE Proc. Sci. Meas. Technol..

[14] Judd M., Yang L., Hunter I. (2005). Partial discharge monitoring for power transformer using UHF sensors. Part I: Sensors and signal interpretation. IEEE Electr. Insul. Mag..

[15] Lee C.H., Lin Y.C., Chiu M.Y., Huang C.H., Yen S.S., Haeng C. The study on diagnostics for aging trend of cable termination. Proceedings of the International Conference on Condition Monitoring and Diagnosis.

[16] Judd M., Farish O., Pearson J.S., Hampton B.F. (2001). Dielectric Windows for UHF Partial Discharge Detection. IEEE Trans. Dielectr. Electr. Insul..

[17] Judd M. Locating Partial Discharges in Power Transformers. Proceedings of the 10th Euro TechCon.

[18] Hoshino T., Nojima K., Hanai M. (2004). Real-time PD identification in diagnosis of GIS using symmetric and asymmetric UHF sensors. IEEE Trans. Power Del..

[19] Ju T., Zhongrong X., Xiaoxing Z., Caixin S. GIS partial discharge quantitative measurements using UHF microstrip antenna sensors. Proceedings of the Annual Report on Electrical Insulation and Dielectric Phenomena.

[20] Balanis C. (2005). Antenna Theory–Analysis and Design.

[21] Ahmed O.M.H., Sebak A.R. (2009). A Novel Maple-Leaf Shaped UWB Antenna with a 5.0–6.0 GHz Band-Notch Characteristic. Prog. Electromagn. Res..

[22] Ebnabbasi K. (2013). A bio-inspired printed-antenna transmission-range detection systems. IEEE Antennas Propag. Mag..

[23] Silva P.F., Freire R.C.S., Serres A.J.R., Silva P.H.F., Silva J.C. (2016). Wearable Textile Bioinspired Antenna for 2G, 3G and 4G Systems. Microw. Opt. Technol. Lett..

[24] Serres A.J.R., Serres G.K.F., Silva P.F., Freire R.C.S., Cruz J.N., Albuquerque T.C., Oliveira M.A., Silva P.H.F. (2017). Bio-Inspired Microstrip Antenna. IntechOpen.

[25] Cruz J.N., Serres A.J.R., Oliveira A.C., Xavier G.V.R., Albuquerque C.C.R., Costa E.G., Freire R.C.S. (2019). Bio-Inspired Monopole Antenna Applied to Partial Discharge Detection. Sensors.

[26] Nobrega L.A.M.M., Xavier G.V.R., Aquino M.V.D., Serres A.J.R., Albuquerque C.C.R., Costa E.G. (2019). Design and Development of a Bio-Inspired UHF Sensor for Partial Discharge Detection in Power Transformers. Sensors.

[27] Yang F., Peng C., Yang Q., Luo H., Ullah I., Yang Y. (2016). An UWB Printed Antenna for Partial Discharge UHF Detection in High Voltage Switchgears. Prog. Electromagn. Res. C.

[28] Veselago V.G. (1968). The Electrodynamics of Substances with Simultaneously Negative Values of ε and µ. Sov. Phys. Uspekhi.

[29] Pendry J.B., Holden A.J., Stewart W.J., Youngs I.I. (1996). Extremely Low Frequency Plasmons in Metallic Mesostructures. Phys. Rev. Lett..

[30] Pendry J.B., Holden A.J., Robbins D.J., Stewart W.J. (1999). Magnetism from Conductors and Enhanced Nonlinear Phenomena. IEEE Trans. Microw. Theory Tech..

[31] Shelby R.A., Smith D.R., Schultz S. (2001). Experimental Verification of a Negative Index of Refraction. Science.

[32] Enoch S., Tayeb G., Sabouroux P., Guerin N., Vincent P. (2002). A Metamaterial for Directive Emission. Phys. Rev. Lett..

[33] Ziolkowski R.W., Erentok A. (2006). Metamaterial-Based Efficient Electrically Small Antennas. IEEE Trans. Antennas Propag..

[34] Islam M.T., Islam M.M., Samsuzzaman M., Faruque M.R.I., Misran N. (2015). A Negative Index Metamaterial-Inspired UWB Antenna with an Integration of Complementary SRR and CLS Unit Cells for Microwave Imaging Sensor Applications. Sensors.

[35] Soffiati A., Max Y., Silva S.G., Mendonça L.M. (2018). Microwave Metamaterial-Based Sensor for Dielectric Characterization of Liquids. Sensors.

[36] Majid H.A., Rahim M.K.A., Masri T. (2009). Microstrip Antenna’s Gain Enhancement of Circular Microstrip Antenna with Left-Handed Metamaterial Structure. Prog. Electromagn. Res..

[37] Srivastava K., Kumar A., Chadhary P., Kanaujia B.K., Dwari S., Verma A.K., Esselle K.P., Mittra R. (2018). Wideband and High-Gain Circularly Polarized Microstrip Antenna Design Using Sandwiched Metasurfaces and Partially Reflecting Surface. IET Microw. Antennas Propag..

[38] Das S., Mitra D. (2018). A Compact Wideband Flexible Implantable Slot Antenna Design with Enhanced Gain. IEEE Trans. Antennas Propag..

[39] Singh A.K., Abegaonkar M.P., Koul S.K. (2017). High Gain and High Aperture Efficiency Cavity Resonator Antenna Using Metamaterial Superstrate. IEEE Antennas Wirel. Propag. Lett..

[40] Venkatesan R., Singaravelu R. An Overview of Metamaterials in Biomedical Applications. Proceedings of the Progress in Electromagnetics Research Symposium (PIERS).

[41] Ramakrishna S.A. (2005). Physics of negative refractive index materials. Rep. Prog. Phys..

[42] Ojaroudiparchim N., Sheng M., Pedersen G.F. Low-Profile Fabry-Pérot Cavity Antenna with Metamaterial SRR Cells for Fifth Generation Systems. Proceedings of the 2016 21st International Conference on Microwave, Radar and Wireless Communications (MIKON).

[43] Singh A.K., Abegaonkar M.P., Koul S.K. A Negative Index Metamaterial Lens for Antenna Gain Enhancement. Proceedings of the 2017 International Symposium on Antennas and Propagation (ISAP).

[44] Paleti S., Bhattacharjee A. Design of PIFA Antenna with Metamaterial Superstrate at 2.4 GHz Using HFSS. Proceedings of the International Conference on Communication and Signal Processing.

[45] Priyadharisini S.G., Rufus E. A Double Negative Metamaterial Inspired Miniaturized Rectangular Patch Antenna with Improved Gain and Bandwidth. Proceedings of the Progress in Electromagnetics Research Symposium (PIERS).

[46] Alhawari A.R.H., Ismail A., Mahdi M.A. Compact Ultra-Wideband Metamaterial Antenna. Proceedings of the Asia-Pacific Conference on Communications (APCC).

[47] Kumar U., Upadhyay D.K., Shahu B.L. Improvement of performance parameters of rectangular patch antenna using metamaterial. Proceedings of the IEEE International Conference on Recent Trends in Electronics Information Communication Technology.

[48] Smith D.R., Padilla W.J., Vier D.C., Nemat-Nasser S.C., Schultz S. (2000). Composite Medium with Simultaneously Negative Permeability and Permittivity. Phys. Rev. Lett..

[49] Ziolkowski R.W. (2003). Design, Fabrication, and Testing of Double Negative Metamaterials. IEEE Trans. Antennas Propag..

[50] Islam M.T., Ashraf F.B., Alam T., Misran N., Mat K.B. (2018). A Compact Ultrawideband Antenna Based on Hexagonal Split-Ring Resonator for pH Sensor Application. Sensors.

[51] Pandit S., Mohan A., Ray P. (2017). A Low-Profile High-Gain Substrate Integrated Waveguide Slot Antenna with Suppressed Cross-Polarization using Metamaterial. IEEE Antennas Wirel. Propag. Lett..

[52] Zheng Y., Gao J., Zhou Y., Cao Z., Yang H., Li S., Li T. (2017). Wideband Gain Enhancement and RCS Reduction of Fabry-Perot Resonator Antenna with Chessboard Arranged Metamaterial Superstrate. IEEE Trans. Antennas Propag..

[53] Hashemi S.K. (2011). Microwave Devices and Techniques for Ultra Wideband (UWB) Communication Systems. Ph.D. Thesis.

[54] Smith D.R., Schultz S., Markos P., Soukoulis C.M. (2002). Determination of effective permittivity and permeability of metamaterials from reflection and transmission coefficients. Phys. Rev. B.

[55] Jaber A.A., Lazaridis P.I., Moradzadeh M., Glover I.A., Zharis Z.D., Vieira M.F., Judd M.D., Atkinson R.C. (2017). Calibration of Free-space Radiometric Partial Discharge Measurements. IEEE Trans. Dielectr. Electr. Insul..

[56] Bartnikas R., McMahon E. (1979). Engineering Dielectrics 1: Corona Measurement and Interpretation.

[57] Ogihara H. (1964). Detection and location of coronas in oil immersed transformers with corona detector. Electr. Eng. Jpn..

[58] International Electrotechnical Commission (IEC) (2011). Power Transformer–Part 1: General.

[59] Wang M., Vandermaar A.J., Srivastava K.D. (2002). Review of condition assessment of power transformers in service. IEEE Electr. Insul. Mag..

[60] Jaber A., Lazaridis P., Saeed B., Zhang Y., Khan U., Upton D., Ahmed H., Upton D., Mather P.J., Atkinson R.C. Frequency spectrum analysis of radiated partial discharge signals. Proceedings of the EUROEM 2016.

[61] Chai H., Phung B.T., Zhang D. Development of UHF Sensors for Partial Discharge Detection in Power Transformer. Proceedings of the 2018 Condition Monitoring and Diagnosis (CMD).

[62] Zhang Y., Lazaridis P., ABD-Alhameed R., Glover I. (2017). A compact wideband printed antenna for free-space radiometric detection of partial discharge. Turk. J. Electr. Eng. Comput. Sci..

[63] Mukhtar S.M., Isa M., Al-Hadi A.A. (2018). Design of UHF Antenna Sensor for Partial Discharge Detection in High Voltage Substation. IOP Conf. Ser. Mater. Sci. Eng..

[64] Wang Y., Wu J., Chen W., Wang Y. (2014). Design of a UHF Antenna for Partial Discharge Detection of Power Equipment. J. Sensors.

[65] Zhang Y., Glover I. Design of an Ultrawideband VHF/UHF Antenna for Partial Discharge Detection. Proceedings of the 2014 IEEE International Conference on Signal Processing, Communications and Computing (ICSPCC).

[66] Marchal A., Monedero M., Le Thuc P., Staraj R. Ultra-wide band antenna for partial discharge detection inside switchgear for on-line monitoring. Proceedings of the 2018 IEEE Conference on Antenna Measurements & Applications (CAMA).

[67] Bin L., Jingle A., Weidong Z., Youlin X. (2013). A Design of Multi-band UHF Sensor for Partial Discharge Detection. Trans. Tech. Publ. Appl. Mech. Mater..

[68] Liu J., Zhang G., Dong J., Wang J. (2015). Study on Miniaturized UHF Antennas for Partial Discharge Detection in High-Voltage Electrical Equipment. Sensors.

[69] Ye H., Qian Y., Dong Y., Sheng G., Jiang X. (2014). Development of multi-band ultra-high-frequency sensor for partial discharge monitoring based on the meandering technique. IET Sci. Meas. Technol..

[70] Li J., Wang P., Jiang T., Bao L., He Z. (2013). UHF stacked Hilbert antenna array for partial discharge detection. IEEE Trans. Antenn. Propag..

[71] Yao C., Chen P., Huang C., Chen Y., Qiao P. (2013). Study on the Application of an Ultra-High-Frequency Fractal Antenna to Partial Discharge Detection in Switchgears. Sensors.

[72] Yongqiang W., Zhuang W., Jianfang L. (2017). UHF Moore Fractal Antenna for Online GIS PD Detection. IEEE Antennas Wirel. Propag. Lett..

[73] Lopez-Roldan J., Tang T., Gaskin M. (2008). Optmisation of a sensor for onsite detection of partial discharges in power transformers by the UHF method. IEEE Trans. Dielect. Electr. Insul..

[74] FT-RF 500 MHz to 8 GHz Double Ridged Broadband Waveguide Horn Antenna. Model: HA-05M08G-NF. http://www.ft-rf.com/front/bin/ptdetail.phtml?Part=500MHz-8GHz-Double-Ridge-Broadband-Antenna-N-Female&Category=359789.

[75] EC Microwave Broadband Horn Antenna Operating From 500 MHz to 3 GHz Double Ridged. http://www.ecmicrowave.com/m_product/96-Broadband-Horn-Antenna-Operating-from-500MHz-to-3GHz-Double-Ridged.html.

[76] Bancroft R., Chou R.C. (2013). Vivaldi Antenna Impedance Bandwidth Dpendence on Stripline to Bilateral Slotline Transition. Microwave Opt. Technol. Lett..

[77] Biancheri-Astier M., Diet A., Le Bihan Y., Grzeskowiak M. (2019). UWB Vivaldi Antenna Array Lower Band Improvement for Ground Penetrating Radar Applications. Radioengineering.

[78] Aaronia AG Active Highhend LogPer Antenne HyperLOG 30100X. https://www.aaronia.com/products/antennas/HyperLog-30100-X/.

